# Influence of Different Light Spectrums on Behaviour and Welfare in Laying Hens

**DOI:** 10.3390/ani11040924

**Published:** 2021-03-24

**Authors:** Anette Wichman, Rosan De Groot, Olle Håstad, Helena Wall, Diana Rubene

**Affiliations:** 1 Department of Animal Environment and Health, Swedish University of Agricultural Sciences, 750 07 Uppsala, Sweden; rosandegroot@gmail.com; 2 Department of Anatomy, Physiology and Biochemistry, Swedish University of Agricultural Sciences, 750 07 Uppsala, Sweden; Olle.Hastad@slu.se; 3 Department of Animal Nutrition and Management, Swedish University of Agricultural Sciences, 750 07 Uppsala, Sweden; Helena.Wall@slu.se; 4 Department of Crop Production Ecology, Swedish University of Agricultural Sciences, 750 07 Uppsala, Sweden; Diana.Rubene@slu.se

**Keywords:** poultry, light, behavior, welfare, *Gallus gallus*

## Abstract

**Simple Summary:**

This study investigated how different types of lighting affect laying hen behaviour and welfare. Amount and quality of light are important for birds to perform their natural behaviours, e.g., find food and water, recognise conspecifics and safely navigate their environment. The lighting used in poultry production facilities differs considerably from light conditions in the natural environment in which domestic fowl have evolved, which might have negative consequences for their welfare. This study examined whether light closely resembling natural daylight and light found in forest understory in Southeast Asia (ancestral habitat of jungle fowl) affected the behaviour of laying hens. The results revealed that birds had a preference for natural lighting in some situations. It is likely that these effects were due to the presence of ultraviolet light, which is known to be important for visual performance in birds. However, the differences were rather small, indicating that sufficient light intensity and other quality factors in the housing environment are more important in maintaining high welfare than the specific spectral composition of the light. This new knowledge can be used to improve the housing environment of domestic fowl by supplying artificial spectrums replicating natural lighting.

**Abstract:**

Artificial commercial lighting used in animal production facilities can have negative influences on visual abilities, behaviour and welfare of domestic fowl. This study examined the effects of natural-derived light spectrums on behaviour, production and welfare of laying hens reared from hatching into adulthood. Comparisons were made of frequency of a range of behaviours associated with activity, aggression and comfort in birds kept in control light (commercial standard), daylight (full spectrum, including ultraviolet (UV) wavelengths and forest light (forest understorey, including UV). In addition, bird preferences for different lights, feather damage and egg production were monitored. The results showed that the behavioural repertoire of birds changed with age, while the effects of light treatment were subtle. Some evidence was found that birds preferred either daylight or forest light to control light, suggesting that inclusion of UV contributed to the preference. Daylight and forest light were associated with more active behaviours, and daylight with better plumage and later start of lay. Thus natural-like light may have beneficial effects on domestic fowl, but the differences between broad-spectrum light sources are rather small.

## 1. Introduction

Vision is one of the most important senses in fowl and, since they are active during daytime, light has a large influence on their behaviour [1]. Laying hens used for commercial purposes are often kept in an artificial light environment. In order to achieve good welfare under these conditions, the type and intensity of light provided must suit the visual needs of the hens [2]. While awareness of poultry welfare issues is increasing, there is still a lack of knowledge on how to optimise the light environment to increase welfare.

The domestic hen originates from the red jungle fowl (*Gallus gallus*), which inhabits a variety of forest types in south-east Asia. It prefers dry deciduous or bamboo forests, forest roads and clearings, or secondary forest near human settlements, where it forages in open habitats during day and roosts inside the forest at night [3,4]. In the forest, the light is filtered through the green vegetation and is different from the light conditions in open areas [5]. Natural light contains a much broader spectral range than artificial indoor lighting and includes ultraviolet-A (UVA) radiation. UVA radiation is also present beneath forest canopies. Fowl have well-developed colour vision and are able to see within the UV range, which probably provides them with better ability to find foods such as berries, seeds and insects that reflect UVA [6]. Parts of the plumage of fowl also reflect UV light, which might have an impact on peer recognition and social encounters [7] and influence mating behaviour [8]. Therefore, UV light could be expected to be required for fowl to perform the full range of their natural behaviours. 

Laying hens are increasingly being housed in aviaries instead of cages, with more space and possibilities for a larger behavioural repertoire. However, problem behaviours such as feather pecking (i.e., when birds pull out the feathers of other birds) still occur in these systems, and the consequences of feather pecking can be severe in large flocks. The causes of feather pecking are multifactorial, e.g., access to litter, genetics, nutrition [9] and overall level of stress affect this behaviour [10]. Providing birds with inappropriate light conditions can be an additional factor that leads to stress and increases the risk of birds developing feather-pecking behaviour [11]. Basal corticosterone levels are higher in chicks reared in a UV-deficient environment than in chicks supplied with UV [12] and layers provided with additional UV have lower stress and fear levels [13]. However, while turkeys show reduced levels of injurious pecking under UV light [14], brown layers under commercial conditions have been found to have more skin injuries and plumage damage when housed with additional UV light [15]. No effects on feather pecking of other colours of light (red, green and blue) have been found as far as we know [16]. It is currently unclear whether the contradictory effects of UV light depend on the intensity of light provided, deviation from natural light, spectral composition or other factors in the bird’s environment.

Other behaviours and production traits in fowl may also be influenced by different light colours. For instance, red light can increase activity level [17] and fear in broilers [18], while in layers it is linked to an increase in productivity [16,19]. A combination of red and white light appears to reduce stress levels in layers [20]. However, contradictory results have been found in broilers, with, e.g., “cool” light (more blue) being more beneficial in reducing stress levels than “warm” light (more red/yellow) [21]. Blue light is also associated with an increase in perching behaviour [16], while green light may be associated with lower mortality and higher fertility [22]. 

Given a choice, birds could be expected to choose a light environment that is most suitable for their biological needs and this preference could be expected to lead to higher welfare [23]. Previous studies have found a general preference for blue and green light in broilers [17], which also show a slight preference for broad-spectrum light that resembles natural daylight (contains more blue and green than red light) [24,25]. Broilers also perform more resting and comfort behaviours in this type of light and gain more weight [25]. Thus, research findings regarding effects of different colours of light on fowl sometimes point in different directions. In general, however, it seems that fowl are calmer and show a preference for light environments with more blue and green colours, and UV can be an important component of the light. This suggests that light conditions resembling natural light could be beneficial for the welfare of fowl. However, there is still a lack of knowledge on the long-term effects (from rearing to laying) of natural light spectrums on the behaviour and welfare of laying hens.

This study evaluated the effect of natural light spectrums, derived based on jungle fowl habitat, on laying hen welfare from rearing until adulthood. Two types of natural light spectrums—daylight (combination of UV and white) and forest light (combination of UV, blue, green and red based on light measurements in South Asia)—were compared with the type of white light without UV conventionally used in poultry housing. The hypothesis was that the visual system of laying hens is still adapted to the type of light in which they evolved, i.e., the light spectrum in their ancestral environment. Considering this hypothesis together with findings from previous studies [24,25], a light spectrum that resembles the natural habitat of the fowl should lead to lower stress levels and lower incidence of feather pecking. To examine this hypothesis, the birds’ preference for, and behaviour in, the different light treatments were observed from the age of 6 to 21 weeks, and data on health and performance were collected. 

## 2. Material and Methods

### 2.1. Animals and Housing

A total of 192 white laying hens of the hybrid Bovans Robust were used as experimental animals. They were bought from a commercial hatchery and arrived as day-old chicks at the Swedish Livestock Research Center at Lövsta, Uppsala. The chicks were vaccinated against Marek’s disease at hatch and against coccidiosis at one week of age. The pens measured 3.56 × 3.62 m (total area 13.4 m^2^), of which around one-third was litter area and two-thirds raised slatted floor. Two group nests (1.15 × 0.46 m each) that were closed off during rearing were opened at the start of lay. Three perches (2.15 m each) were provided at different heights over the slatted floor. The chicks were initially kept in the litter area in six groups, with 32 chicks per group. A heating lamp provided additional warmth (around 37 °C) to the chicks in the first weeks. The general room temperature was initially 23–24 °C and was then gradually reduced to 20 °C by week 5. When the chicks were 18 days old, they were allowed access to the slatted floor, while feeders and drinkers were moved up onto this area at 25 days of age. Wood-shavings were provided as litter and the birds had ad libitum access to feed and water from round feeders and bell drinkers adapted in height to the size of the birds. The chicks were fed commercial full feed adapted to their age, i.e., starter feed until week 5, grower feed until week 17 and layer feed from then until the study ended when the birds were 27 weeks old.

### 2.2. Ethical Considerations

The regional ethics committee for animals used for scientific purposes in Uppsala, Sweden, approved the study according to C79/16, and studies were performed in accordance with Swedish animal welfare legislation. The animals were checked twice daily as chicks and at least once daily as pullets and adults. Birds showing signs of sickness or injuries were euthanized, treated with anti-peck spray (No fight; Kerbl, Buchbach, Germany) or moved to a separate pen for recovery, depending on the cause and health status of the bird. The observations and treatments performed in the study were not expected to cause harm or stress to the birds. When birds needed to be handled, this was done carefully to minimise potential stress. The chicks were habituated to the presence of a human in their home pens for 10 min per day during the first weeks of their life. At termination of the study, most of the hens moved on to private homes.

### 2.3. Light Treatments

The light spectrum was measured using a spectrophotometer (Jaz A3420, Ocean Optics, Dunedin, FL, USA) at two sites in the district of Tamil Nadu in SE India: secondary forest/rural settlements in Auroville (12.006944° N, 79.810556° E, WGS84, elevation 30 m) and natural dry deciduous forest on a hillslope in Yercaud, Eastern Ghats (11.77599° N, 78.20927° E, WGS84, elevation 500–700 m). The forests contained tropical dry deciduous or tropical dry evergreen vegetation [26], with a considerable abundance of bamboo, which is typical of the jungle fowl habitat [3,4]. At each site, light spectrum was measured in: (a) forest interior (to represent roosting habitat) and (b) forest edge (to represent foraging habitat) in clear or partly cloudy weather conditions. Light measurements were taken at 0° and 90° relative to the ground, about 20 cm above the ground, at midday and in mornings just after sunrise and evenings just before sunset on three days at each site. Several measurements were made on each visit, at 5–20 min intervals, as the light spectral composition changed during dawn and dusk [5]. However, the light levels at dawn and dusk were too low to collect measurements, particularly from the forest interior, so no clear differences in the light spectrum were observed between morning/evening and midday sessions. Two characteristic types of spectrums were obtained, for forest understory and forest edge respectively, and the individual measured spectrums at the different sites were highly similar within these two types. The forest interior was dominated by green light, while the forest edge had more blue wavelengths, which is in accordance with types of light spectrums measured previously in forest habitats [5]. Spectrums from forest understory were used as the basis for a ‘Forest light’ treatment. Spectrums from forest edge closely matched that of average daylight spectrum, D65 (DIE Standard Illuminant), which was used as the basis for a ‘Daylight’ treatment. 

In order to match the experimental lights to the natural spectrums perceived by the visual system in fowl, they were calibrated by calculating the quantum catch for each of the single cones of the chicken eye, using MATLAB (R2014b, MathWorks Inc., Natick, MA, USA). The measured natural spectrums between 300 and 750 nm were averaged, converted to photons/m^2^/nm and multiplied by the single-cone relative spectral sensitivity of *Gallus gallus* [27,28], as previously described in Rubene et al. [29]. Relative proportion of the light absorbed by each type of single cone receptor for each spectrum (forest and daylight) was then calculated.

**Figure 1:**
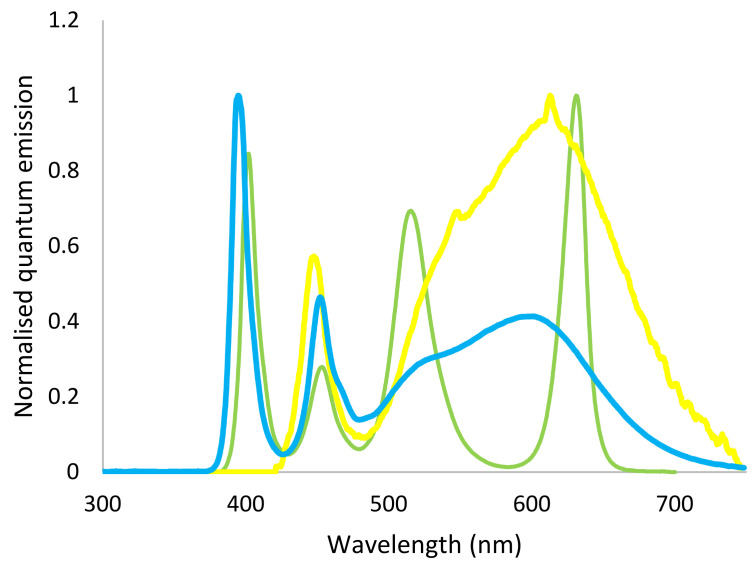
Spectral composition of the three light treatments used in the study: Daylight (**blue**), Forest (**green**) and Control (**yellow**).

Experimental lights were constructed to match the relative cone stimulus of the light from the field measurements. For Daylight (Figure 1) LEDs with white (4500 K) and UV (peak 395 nm) spectrums were matched as closely as possible to the UV:white ratio of D65. Forest light (Figure 1) was built using RGB LEDs (red peak 630 nm, green peak 515 nm, blue peak 453 nm) and UV LEDs, where the proportion of UV and the red:green:blue ratio were adjusted to match the forest understory spectrums measured in SE India, using the same calculation procedure as described above. Control light (Figure 1) with a warm white spectrum (approximately 3000 K) and containing no UV was provided by custom-made lamps already in use in the house. In order to ensure that the light intensity was similar across treatments, it was measured at five different locations at bird level in each pen (four corners and centre) twice during the study period, using a light meter (Mastech MS6610, Wanchai. Hong Kong). Mean light intensity in the treatments was: Control 7.9 ± 1.0 lux, Daylight 10.9 ± 1.4 lux and Forest light 8.3 ± 0.05 lux.

The chicks were all reared in Control light until 5 weeks of age, when each initial group of 32 chicks was divided into two groups, so that there were a total of 12 groups with 16 birds in each. These were allocated to the three different light treatments (Forest, Daylight and Control), with four groups per treatment. All pens were located in two rows in the same room. To prevent light from the different light treatments entering the other pens, grey sheets of tarpaulin were placed between pens, but leaving some space under the roof to allow for ventilation. Pens located opposite each other had the same light treatment but pens next to each other had different treatments, to allow for a balanced distribution of light treatments in the room. The light schedule followed recommendations from the hatchery during the first rearing period, with lights on for 23 h at start and gradually decreased to 10 h at 49 days of age. The light period was increased by one hour at 15 and 16 weeks of age, respectively. Twelve hours of light (06.00–18.00 h) were then maintained for the rest of the study, to give the same hours of daylight throughout the preference tests. 

### 2.4. Behaviour Observations in Home Pen

Observations of the birds’ behaviour in the home pen were carried out at 8, 12, 16 and 21 weeks of age. Instantaneous and continuous observations of all birds were made in each pen in the morning and afternoon. During the continuous observations, the frequency of the different behaviours performed was counted for 10 min. For the instantaneous observations, behaviours performed at three specified time points (before, during (after 5 min) and after the continuous observations) were recorded. The continuous observations focused on social behaviours like feather pecking and aggression, whereas the instantaneous observations focused on behaviours usually performed over a longer time (Table 1). The observer was located outside the pen but visible to the birds, so that they could adjust to the presence of the observer for a couple of minutes before the observations began. Two observers carried out the observations and agreement between observers was compared before the first observations were carried out. 

### 2.5. Preference for the Different Light Environments

The birds’ preference for the different light environments was investigated at 16–24 weeks. Three separate test pens of the same size and layout as the home pens were used. The pens were divided into two similar sections by tarpaulin that hung from the ceiling to 30 cm above the floor. The two sections were similarly equipped with feeder, drinker, nest box, perches and litter area. Different light treatments were set up in each section of each test pen, in combinations Control + Daylight, Control + Forest and Forest + Daylight. Birds from each home pen were moved as a group to the test pens and exposed to the test three times, for three days at a time, and once with each light combination. The order of testing was balanced between groups and each group was tested once in each test pen. The allocation of light treatment within and between pens was also changed between tests, to avoid any spatial preference bias. Instantaneous observations were carried out six times, with approximately 5 min in between each observation, during four periods each day, starting at 8.30, 11.30, 14.30 and 17.30 h. Observations followed a similar protocol as in the home pen, but location of the bird was also noted, according to the following:

Perch: standing, locomotion, resting, preening
Slats: feeding, drinking, standing, locomotion, resting, preening
Litter: foraging, standing, locomotion, resting, comfort

### 2.6. Body Weight, Plumage and Integument Assessment

All birds were weighed at 5 and 25 weeks of age. In combination with final weighing, feather and integument scoring were carried out on each bird. Four areas of the bird were scored (body, tail, comb and feet), using the scoring protocol developed by Bilcik and Keeling [30] (Table 2), but using only one overall score of the whole body. The tail was scored separately, as flight feathers, while the comb and feet were scored according to the protocol for skin injuries. 

### 2.7. Egg Production and Mortalities

Eggs were collected daily and counted for each pen. Mortalities and possible cause were also noted when these occurred, but no autopsy was performed on the deceased birds.

### 2.8. Statistical Analyses 

*Behaviours in home pen*: Home pen behaviours observed continuously (aggression, gentle pecking, wing-shaking, stretching, runs, sparring and vocalisation) were analysed with the Glimmix procedure in SAS (SAS Institute Inc. version 9.4, Cary, NC, USA). Included in the model as fixed factors were light and week (to test for age effect) and the interaction between these. The data were blocked by pen (random factor) which was the statistical unit (*n* = 12) and repeated by week. For the response variables aggression, gentle pecking, wing shaking, stretching and vocalisation, Poisson distribution with a log link was used, since the data were not normally distributed. The number of birds was included as an offset to account for the different number of birds in some of the pens. For the response variables runs and sparring, normal distribution was assumed due to the convergence criterion not being satisfied with a Poisson distribution. Analyses were carried out on mean per pen and on mean per week for the different weeks in which the birds were observed. A Tukey adjustment was included and post hoc data are based on the adjusted means. Regarding the behaviours recorded as scans (comfort behaviour, locomotion, perching, standing, foraging, drinking and feeding), these were analysed with Glimmix in SAS with Poisson distribution. Number of birds was included as an offset to account for the different number of birds in some of the pens. 

*Preference for different light treatments*: The preference for different light treatments was analysed in the SAS Glimmix procedure with binomial distribution, both regarding the general preference for the different light environments and for the specific areas birds were observed in (litter, slats and perches) and three categories of behaviour (resting, active and comfort). Active behaviours were foraging, locomotion and standing combined. Fixed factors were test light (in preference pens) and home light, and their interaction. Random factors were group (and round). The model was scaled to correct for overdispersion. The observations were summed per observation period and group before analyses were carried out. That led to one observation point per group for all days (3–4) they were in the same paired choice, with *n* = 4 per home light treatment in each paired preference test. 

To test for preference the first time the hens experienced a new light (one section of the pen contained home light and the other a new light), the number of birds observed in the home light was divided by total number of birds observed, and then the mean per day was calculated per group. These proportions of choice depending on the light in the home pen were subjected to a one sample T-test, assuming the value would be significantly different from 0.5.

*Body weight, plumage and integument assessment*: The data had a normal distribution and analyses were carried out on the mean value for each pen in a general linear model (GLM) with treatment as the only factor both for weight and for feather score data. 

*Egg production*: Egg production was calculated as the percentage of eggs (number of eggs per pen divided by number of birds in the pen) produced per week from start of lay until 26 weeks of age. The data were normally distributed and differences between treatments were analysed with GLM. Analyses on body weight, plumage and egg production were done in SPSS (IBM Statistics version 26, Armonk, NY, USA).

## 3. Results

### 3.1. Behavioural Observations in Home Pen

#### 3.1.1. Effect of Light

There were no significant differences between the light treatments as regards behaviours observed in either continuous or instantaneous observations in the home pens. There were also no significant interactions between light and age (all *p* > 0.05). 

#### 3.1.2. Effect of Age

Age had an effect on the frequency of expression of the behaviours gentle pecking (F_3,27_ = 3.20, *p* = 0.039), wing shaking (F_3,27_ = 5.19, *p* = 0.006) stretching (F_3,27_ = 6.01, *p* = 0.003), vocalisation (F_3,27_ = 7.9; *p* = 0.0007), runs (F_3,27_ = 11.51, *p* < 0.0001) and sparring (F_3,27_ = 4.721, *p* = 0.009). There was no difference between ages for the behaviour aggression (F_3,27_ = 1.85, *p* = 0.16). Severe pecking was only observed 13 times over all observations (5, 2 and 6 times in C, D and F, respectively) and was therefore excluded from the statistical analyses. 

**Figure 2:**
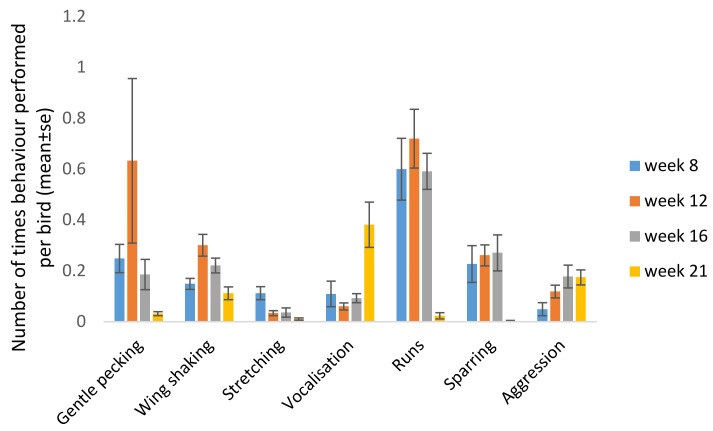
Number of times different behaviours were observed per bird in the home pen at 8, 12, 16 and 21 weeks of age (mean of all light treatments).

More specifically, assessments of the effect of age on performance of different behaviours showed that birds performed less running at 21 weeks of age compared with when they were younger (21 weeks vs. 8, 12 and 16; *p* = 0.0007, *p* < 0.0001 and 0.0009, respectively). There was a similar pattern for sparring (21 weeks vs. 8, 12 and 16; *p* = 0.05, *p* = 0.004 and *p* = 0.015, respectively). Wing shaking was performed more frequently at week 12 compared with weeks 8 (*p* = 0.019) and 21 (*p* = 0.019). Stretching was performed more often at week 8 compared with older ages (vs. week 21 *p* = 0.015), whereas vocalisation was significantly more frequent at week 21 compared with week 8 (*p* = 0.0208), week 12 (*p* = 0.010) and week 16 (*p* = 0.022) (Figure 2).

In the instantaneous behaviour observations in the home pen, effects of age were found for the behaviours locomotion (F_3,26_ = 9.07, *p* = 0.0003), perching (F_3,26_ = 3.77, *p* = 0.023) and resting (F_3,26_ = 13.9, *p* = 0.000), while there was a tendency for a difference in standing behaviour (F_3,26_ = 2.86, *p* = 0.056). No effect of age was found on the behaviours foraging, comfort, drinking and feeding. 

**Figure 3:**
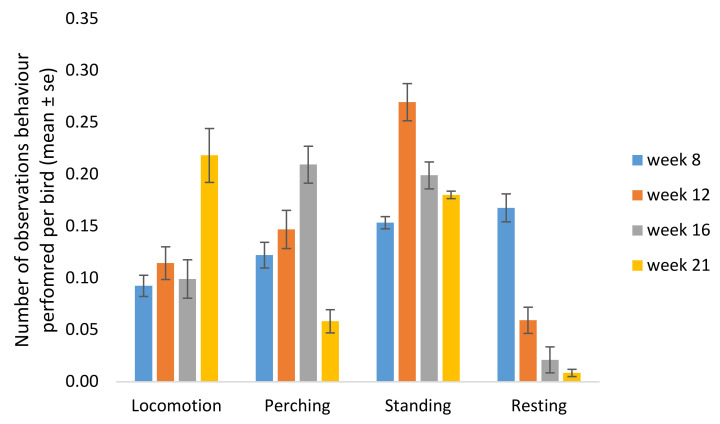
Number of instantaneous observations in which birds performed different behaviours at 8, 12, 16 and 21 weeks of age (mean of all light treatments).

*Locomotion*: Locomotion occurred more often in week 21 compared with week 8 (*p* = 0.0002), week 12 (*p* = 0.0022) and week 16 (*p* = 0.0007), whereas perching increased from week 8 to 16 (*p* = 0.038) but then decreased in week 21 (week 16 vs. 21 *p* = 0.005). Standing occurred more often in week 12 compared with week 8 (*p* = 0.0077). Resting decreased over age and differed between most weeks, and was less frequent in week 21 compared with the other weeks (week 8 vs. 12 *p* = 0.0015, week 8 vs. 16 *p* = 0.00010, week 8 vs. 21 *p* = 0.0004, week 12 vs. 16 *p* = 0.046, week 12 vs. 21 *p* = 0.021) (Figure 3).

### 3.2. Preference for the Different Light Environments

**Figure 4:**
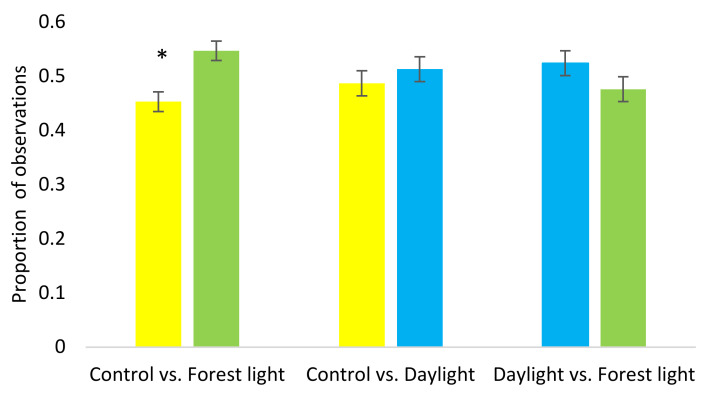
Pairwise comparisons from the preference test of proportion of birds observed in the different sections with different light treatments. Asterisk indicates significant difference (*p* < 0.01) between light treatments.

Results from the preference tests showed a tendency for an overall difference in preference for the different lights (Glimmix; F_2,54_ = 2.95, *p* = 0.061). In general, the birds tended to spend more time in Daylight (*p* = 0.071) compared with Control light. In pairwise comparisons for each of the three different light combinations, significantly more birds were observed in the section with Forest light when the other section had Control light (F_1.22_ = 13.32, *p* = 0.001) (Figure 4), but there were no significant differences between the other light combinations. 

There was no overall significant difference in the birds’ preference for the different light treatments depending on home light (F_2,54_ = 0.01, *p* = 0.995), and no interaction between test light and home light (F_4,54_ = 2.02, *p* = 0.105). However, analyses based on six groups that could choose between home light and a novel test light on their first trial (the other six groups could only choose between two novel lights) indicated a preference for home light on the first day in the preference pen (T-test; *p* = 0.045, 59 ± 3.4 mean ± se%). This preference did not remain on the following days (Day 2; *p* = 0.82, 51 ± 4.7, Day 3 *p* = 0.95, 49 ± 7.1). 

The performance of active behaviours (locomotion, standing and foraging combined) was affected by light (F_2,54_ = 6.38, *p* = 0.0032), with more active behaviours performed in the Forest light and Daylight treatments. There was no effect on resting or comfort behaviours of any of the factors tested.

Comparisons of use of litter, slats or perches showed a significant effect of home light on the use of the litter area (F_2,54_ = 5.92, *p* = 0.0047), with birds from the Daylight (*p* = 0.0042) and Forest light treatments (*p* = 0.067) observed less often in the litter than birds from the Control. In addition, test light tended to influence the birds’ location in the pen (F_2,54_ = 2.72, *p* = 0.075), with more birds observed in the litter area in Forest light compared with Control light (*p* = 0.098). On slats, a tendency for an effect of test light was found (F_2,54_ = 2.71, *p* = 0.076), with more birds found in Daylight and Forest light than in Control light. In addition, a tendency for an interaction between home light and test light (F_4,54_ = 2.25, *p* = 0.076) was observed, with birds from Forest home light observed more on the slats with Forest light than the slats with Control light (*p* = 0.063). There was no significant effect of either test light or home light on perch use. 

### 3.3. Body Weight, Plumage and Integument Assessment

Mean weight (±se) of all experimental birds was 459.6 ± 2.3 g (*n* = 190) at 5 weeks of age and 1633.5 ± 8.9 g at 25 weeks (*n* = 186). There were no differences in weight between groups at the time when they were assigned and moved to the different light treatments (GLM; F_2,9_ = 0.86, *p* = 0.455) or at 25 weeks of age when they had been in the different light treatments for 20 weeks (F_2,9_ = 0.24, *p* = 0.79).

In general, the birds were in good condition and the highest recorded score for plumage and integument assessment was 3. The mean score for body was 0.18, tail 0.87, comb 0.95 and feet 0.39 (a score of 0 indicates no damage, while the maximum damage score was 5 for body and tail and 4 for feet and comb). Thus, both the body score and feet indicated very good condition, while some birds had scruffy tail feathers and minor injuries or scratches on the combs.

**Figure 5:**
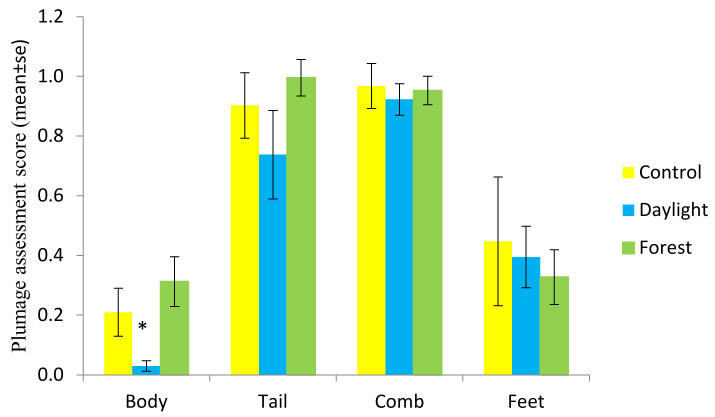
Plumage and integument assessment score (mean ± se) for the body, tail, comb and feet in the different light treatments.

There was a significant difference between treatments for body score (GLM; F_2,9_ = 4.47, *p* = 0.044), with Daylight birds having a better feather score, i.e., lower damage, than Forest light birds (Tukey test *p* = 0.039) (Figure 5). 

### 3.4. Egg Production and Mortality

**Figure 6:**
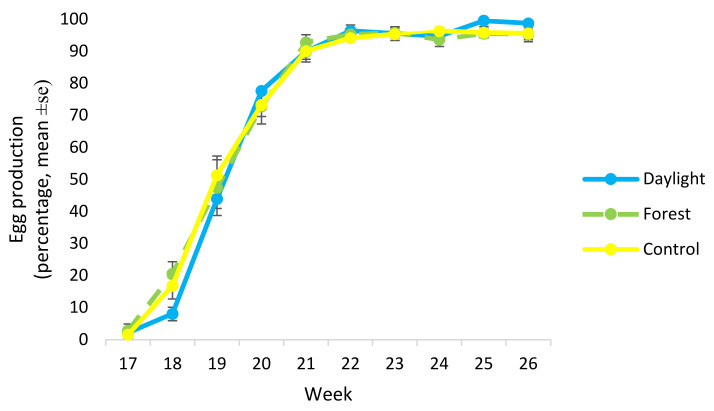
Percentage of eggs produced per week from start of lay until 26 weeks of age by birds from the different light treatments.

There was no difference between light treatments in overall egg production from start of lay at 17 weeks of age until the experiment finished at 27 weeks of age (GLM; F_2,9_ = 0.39, *p* = 0.69). There was a tendency for hens from different light treatments to differ in percentage of lay at 18 weeks of age (GLM; F_2,9_ = 3.40, *p* = 0.079), with the Daylight treatment having a lower percentage of lay compared with the Forest light treatment (*p* = 0.08, Tukey test) (Figure 6).

Four birds died of unknown causes during the study, at 1, 2, 9 and 21 weeks of age, respectively. Two hens from two different control pens were moved to separate pens temporarily due to pecking damage at 23 and 27 weeks of age. 

## 4. Discussion

The ancestor of domestic laying hens, red jungle fowl, evolved in certain light environments, so domestic hens might still be best adapted to these natural light conditions. Birds in this study were expected to show indications of higher welfare and a preference for artificial light spectrums that resembled natural light, rather than the standard white spectrum commonly used in industrial housing. There was some support for the hypothesis that the visual system of laying hens is still adapted to the type of light in which they evolved, since birds tended to prefer the more natural light types (Daylight, Forest) to the white Control light. 

Observations of the birds’ behaviour in the home pens did not show any significant differences between birds reared in the different light treatments. This could be due to all light spectra tested being broad, so that the amount and quality of light were sufficient to provide for the basic visual needs of the birds and enabled them to perform the range of behaviours studied. As expected, differences in behaviours occurred over time, indicating that the observation methods were adequate in detecting differences. Differences may also have been present in other behaviours not assessed in this study, such as behaviours that require high visual accuracy, e.g., social recognition or detection of small food particles. 

The birds’ tendency to preferentially locate themselves in the areas containing the lights that resembled natural light (Daylight, Forest) in the preference test is in agreement with observations in broilers [24,25]. However, the differences in preferences seen in this study, and in studies by Kristensen et al. [24] and Riber [25], were rather subtle, which indicates that the birds did not actively avoid the less natural light environment. The reason for this could be that the contrast between the spectrums used was not large enough to cause the birds to develop strong preferences. Gunnarsson et al. [31] observed a strong preference for incandescent light in chicks that had been reared in incandescent light, whereas birds reared in natural light did not show any preference for either light. This could be due to birds accustomed to natural light experiencing a larger variation in their light environment, and thus adapting more easily to the incandescent light. 

In the preference test, there were indications that either Daylight or Forest light was preferred over Control light, but there was no evidence that the birds had any preference for Daylight over Forest light and vice versa. Both Daylight or Forest light contained UV, but differed in the proportion of blue and green, so it appears that the presence of UV created the difference in preference. Previous studies have shown effects on behaviour following addition of UV to the ambient light, which could be explained by the improved visual feedback this light is supposed to provide for the birds, e.g., effects on specific behaviours such as tendencies for increased exploration [12]. Other general positive effects related to provision of UVA, such as reduced fearfulness in broilers [32] and in layers [12,13,33] have been found. It is not possible to draw any conclusions on exactly how much UV is optimal for laying hens, but proportions matching natural light should be beneficial. 

Regarding the birds’ preference to perform certain behaviours in different light, there were also no differences between Daylight and Forest light, but there was a difference between these and Control light. Active behaviours (locomotion, standing and foraging) were observed more often in Daylight and Forest light than in Control light. This is in agreement with findings by Huber-Eicher et al. [19] that layers perform more foraging behaviour in green light. This could be due to an improvement in the birds’ visual experience in the experimental lights, as activity requires higher visual ability than passive behaviours. In fact, UV can be important for birds’ ability to detect movement [29]. On the other hand, the birds showed no preference for perch use, resting behaviour or comfort behaviours depending on light treatment, which contradicts the expectation that Forest light would be preferred for perching and resting, since more cover might be found in the vegetation. 

When first introduced to the preference testing, the birds initially preferred their home light to a novel light. This effect only lasted for one day, although birds from the Daylight and Forest light treatments preferred to be in the part of the litter area with their respective home light on subsequent days. A short-term preference for a familiar light environment has been found in a previous study on broilers, but the preference was weaker for birds reared in red light compared with blue, and after one week nearly all birds showed a preference for blue light [17]. Differences in preference depending on the spectral properties of light were also observed by Gunnarsson et al. [31] for the first day on which pullets were exposed to a new light environment. This suggests that birds prefer familiarity and can be neophobic to light, as they are to other types of novel environments, but that they can change their preference to other environments. It may even be the case that more subtle spectral differences (two broad-spectrum stimuli) lead to a shorter-lasting preference than differences between spectrally non-overlapping stimuli (like monochromatic red and blue). 

The light treatments did not affect body weight and overall egg production but there was a tendency for Daylight birds to start to lay slightly later. Previous studies have found that exposure to red light can lead to earlier maturity in pullets compared with exposure to green light [34]. There were no differences between Control and Forest light in this study, so the difference seen for the Daylight treatment could be due to the Control light containing relatively more red light than the Daylight and Forest light, with the latter containing most red light. This is in line with previous findings linking red light and longer wavelengths to increased productivity compared with shorter wavelengths [35]. A later start of lay might be positive in the long run, however, as not coming into lay before 20 weeks of age can lead to reduced risk of feather-pecking [36] and improved bone strength [37]. In the present study, the Daylight birds had the best plumage score (although the birds in the Forest and Control light treatments also had nearly perfect plumage). This is possibly in agreement with Green et al. [36], who found that birds coming into lay before 20 weeks of age had an increased risk of feather pecking. However, an alternative explanation for the better plumage score in the Daylight birds could be differences in maturation (regarding start of lay), i.e., that their feathers were in different stages of growth. In addition, problems with feather pecking usually occur in laying hens at ages beyond the 27 weeks examined in this study, so it is difficult to draw any clear conclusions. However, higher frequency of feather-pecking (although not statistically significant) was observed by Hassan et al. [16] in birds housed in monochromatic red light compared with green, blue and fluorescent white light. Since there was no difference in plumage (or start of lay) between Control and Forest light birds, higher content of blue and UV light relative to red light may lead to these differences. There is little direct evidence that the exact light spectrum (given it is fairly broad) would have a large impact on feather pecking, since other environmental requirements of the birds such as availability of litter and forage, good air quality etc. need to be fulfilled. However, if light spectrum can lead to higher well-being of the birds and reduced stress levels, it may have a positive effect on the birds in the long run.

In a study carried out under commercial conditions, brown layers with UV supplementation were found to have worse plumage and more skin injuries than birds housed with standard white light [15]. Similarly, Ruis et al. [33] found an increase in feather pecking in layers with supplemental UV at week 50. In those cases, UV light actually worsened an existing problem, which appears to contradict findings in many other studies, including the present study. It must be borne in mind, however, that commercial conditions are different from the natural environment in many other aspects apart from light quality, e.g., space, flock size, environmental enrichment and light intensity levels. A common management strategy if problems such as feather pecking occur in a flock is to reduce light intensity in order to reduce the visual capacity of birds, and hence their general activity and also feather pecking. Since UV light can improve visual ability, in an already problematic environment it might worsen the problem. Altogether, it seems that appropriate lighting can be beneficial for birds, but it cannot compensate for other shortcomings in their environment. 

## 5. Conclusions

This study showed that laying hens reared and kept into adulthood in different light environments tended to prefer light spectrums that resembled natural light and contained relatively more UV, blue and green than standard lighting. However, they did not seem to have any aversion or display negative behavioural effects when exposed to the standard light, perhaps because of overall high quality of the housing environment. These results add knowledge and support findings from other studies. They also show that such effects persist in layers well into the laying period. However, the positive effects were rather subtle and the birds did not show a preference for control light over forest light or daylight for any of the behavioural factors studied. There is still a need to test natural light spectrums over a longer period and under different light intensities in commercial conditions before recommendations for best practice can be delivered. There is also a need to identify the influence of UV light on the positive experience of light spectrum in laying hens and to determine how best to provide poultry with additional UVA. 

## Figures and Tables

**Table 1:** Ethogram of behaviours observed in the home pens and their description, divided into behaviours observed with instantaneous sampling and with continuous sampling.

**Instantaneous Sampling**	
Drinking	Beak in or above drinker
Feeding	Beak in or above feeder
Standing	Abdomen not touching the litter + bird motionless
Locomotion	Walking (moving legs forward with one leg in contact with floor), or moving between different levels (slats, litter and perches)
Foraging	Pecking or scratching the litter
Resting	Resting abdomen on the litter
Perching	Located on the perch
Comfort behaviour	Preening (beak touches plumage of bird itself) and dustbathing (lying down, scratching and or rubbing litter into the plumage) combined
**Continuous Sampling**	
Aggression	Frontal displays with raised hackles towards other birds, head pecking, jumping or kicking at other bird
Severe pecking	Hard and fast pecks and/or pulling at other birds’ feathers
Gentle pecking	Light, repeated pecks at the feathers of another bird
Stretching	Either wing or leg is lifted off ground and away from body as far as possible
Wing shaking	Both wings are lifted in upward movements
Runs	Moving faster than walking pace, both feet leave the ground in each step
Sparring	Frontal displays, often accompanied by little jumps
Vocalisation	Louder vocalisation like gakel call or short loud sound

**Table 2:** Protocol used for integument and plumage scoring.

Scheme	Body	Flight Feathers (Tail)	Skin Injuries (Comb and Feet)
0	Intact feathers	Intact feathers	No injuries or scratches
1	Some feathers scruffy, up to 3 missing feathers	Few feathers separated but none broken or missing	<5 pecks or scratches
2	More damaged feathers, >3 feathers missing	A lot of feathers separated and/or a few broken or missing	5 or more pecks/scratches or 1 wound < 1 cm diameter
3	Bald patch < 5 cm diameter or < 50% of area	All feathers separated, a lot of broken or missing feathers	Wound >1 cm in diameter but < 2 cm
4	Bald patch > 5 cm diameter or > 50% of area	Most of the feathers missing or broken	Wound > 2 cm in diameter
5	Completely denuded area	Almost all feathers missing	-

## Data Availability

The data presented in this study are available on request from the corresponding author.
